# The commercial impact of pig *Salmonella* spp. infections in border-free markets during an economic recession

**DOI:** 10.14202/vetworld.2015.257-272

**Published:** 2015-03-05

**Authors:** G. Evangelopoulou, S. Kritas, G. Christodoulopoulos, A. R. Burriel

**Affiliations:** 1 Laboratory of Microbiology and Parasitology, Faculty of Veterinary Medicine, School of Health Sciences, University of Thessaly, Karditsa, Greece; 2 Department of Microbiology and Infectious Diseases, Faculty of Veterinary Medicine, Aristotle University, Thessaloniki, MKD, Greece; 3 Department of Clinical Veterinary Medicine, Faculty of Veterinary Medicine, School of Health Sciences, University of Thessaly, Karditsa, Greece

**Keywords:** control, economic crisis, pig, *Salmonella*, salmonellosis

## Abstract

The genus *Salmonella*, a group of important zoonotic pathogens, is having global economic and political importance. Its main political importance results from the pathogenicity of many of its serovars for man. Serovars *Salmonella* Enteritidis and *Salmonella* Typhimurium are currently the most frequently associated to foodborne infections, but they are not the only ones. Animal food products contaminated from subclinically infected animals are a risk to consumers. In border free markets, an example is the EU, these consumers at risk are international. This is why, economic competition could use the risk of consumer infection either to restrict or promote free border trade in animals and their products. Such use of public health threats increases during economic recessions in nations economically weak to effectively enforce surveillance. In free trade conditions, those unable to pay the costs of pathogen control are unable to effectively implement agreed regulations, centrally decided, but leaving their enforcement to individual states. Free trade of animal food products depends largely on the promotion of safety, included in “quality,” when traders target foreign markets. They will overtake eventually the markets of those ineffectively implementing agreed safety regulations, if their offered prices are also attractive for recession hit consumers. Nations unable to effectively enforce safety regulations become disadvantaged partners unequally competing with producers of economically robust states when it comes to public health. Thus, surveillance and control of pathogens like *Salmonella* are not only quantitative. They are also political issues upon which states base national trade decisions. Hence, the quantitative calculation of costs incurring from surveillance and control of animal salmonelloses, should not only include the cost for public health protection, but also the long term international economic and political costs for an individual state. These qualitative and qualitative costs of man and animal *Salmonella* infections should be calculated in the light of free trade and open borders. Understandably, accurate calculation of the economic and political costs requires knowledge of the many factors influencing nationally the quality and safety of pork products and internationally free trade. Thus, how *Salmonella* pig infections affect commerce and public health across open borders depends on a state’s ability to accurately calculate costs for the surveillance and control of animal salmonelloses in general, and pig infections as a particular example.

## Introduction

*Salmonella* spp., a pathogenic genus of Enterobacteriaceae, have serovars causing from subclinical to severe clinical infections in animals and typhoid fever and severe diarrhea in humans [1]. Foodborne *Salmonella* infections are equally important worldwide, similar to those of *Campylobacter* spp., and pork is one of the important sources of pathogenic to man serovars [2,3]. Serovars Enteritidis and Typhimurium have become the most prevalent, replacing previously important serovars [3-5]. The reported prevalence of *Salmonella* spp., although varied among European Member States, implicates pork meat as a potential risk to human health [6,7]. About 93.8 million cases of foodborne salmonellosis occur annually worldwide [8], costing the various national health authorities a mean above US$1000 per case [9,10]. Costs for treating human cases are the actual incentives for some nations, such as Denmark, Sweden, Germany, Belgium and others in undertaking control measures, aiming at the reduction of *Salmonella* prevalence in their pig farms. They are also states inspiring to internationally promote as safe their national products. Their programs have shown that reductions in human *Salmonella* prevalence result from reductions in various points of food production of animal origin and poultry and pork meat are their targets [6,7,11-13].

Such states, members of open border unions, have influenced and will continue influencing the making of uniformed regulations for food safety across borders and examples are various EU regulations [14-16] for effective control pathogens such as *Salmonella* to each member state, thus each state’s ability to bear the costs incurred [6,11-13]. Such programs are, suppose easily adjusted to each country’s burden of disease. They emphasize the benefits to meat producers, if a state can economically subsidize a national control program [7]. However, if a state cannot for economic reasons enforce such community regulations, perhaps due to a recession, a nation’s food chain is exposed to an unfair open border competition on issues of safety. In a union such as the EU (and others to come), where suppose reciprocity is the building block, measures for enforcing community regulations should be incentives, centrally managed and financed, rewarding pig farmers for producing *Salmonella* free pork. Otherwise, winners are big producers, national or international, forcing smaller holdings to a final closing down by offering consumers good prices and safer product. Safety food regulations are in free trade areas supranational and based on punishments; rewards for compliance are national choices. If a nation cannot protect the interests of its food producing business, others will dominate it, perhaps, with long-term political consequences for the receiving economy. Thus, the decision on the form and actual size of rewards to regulated farmers is a political decision influenced by economic and political interests, many of which are passing national borders. In international commerce conditions, economic recessions give space to international competitors able to absorb losses from lower prices [17,18].

Therefore, for small economies, the national final cost of inaction on food safety could be much larger than the actual costs of action. For the protection of national interests, state authorities and pork producers should aim for a consensus on safety avoiding the long-term costs of state inaction. Availability and accumulation of capital is not just an individual responsibility, when it comes to national products, upon which depends on the survival of a nation’s population in times of expected turmoil and economic insecurity [2,19-21]. They are a state priority if national security is the objective of its politicians. However, in the name of economic and political unity of culturally and economically different states, such as in the EU, food safety regulations could increase trading gaps between partners. In such a union, the economic inability to control animal pathogens increases trade inequality if recessions do not promote reciprocity for market harmonization. Thus, lack of reciprocity is one of the many reasons small economies in large unions should subsidize their food production to remain free of important pathogens.

## Reasons for Controlling *Salmonella* spp. Infections in Pig Production

The impact of *Salmonella* spp. control on public health, evidenced in many EU nations and elsewhere by continuous epidemiological surveillance in man and animals [2,3,8,22], is a paradigm on the benefits gained when rewards are offered to farmers for producing *Salmonella* free pork [23]. *Salmonella* prevalence across a border free EU is influenced by freely traded live pigs, pig meat and pork products, national consumption habits and the type of a pig farm management [24]. Consumption of undercooked pork and cross-contamination of consumer products during processing of pork products are high-risk factors [25]. Thus, the protection of public health depends on reliable rules of monitoring and reporting animal *Salmonella* infections and retailing facilities contamination, but also the undertaking of educational programs for increasing the awareness of food workers and consumers on food safety during food preparation [5,22,23].

The worldwide estimated human loss due to salmonellosis is about 155,000 deaths per year, in their majority children up to 4 years of age and mostly caused by serovars Enteritidis and Typhimurium. In the EU, a drop in confirmed human cases was observed the past 5 years of data collection. This reduction was greater between 2008 and 2010, with a drop in confirmed human cases from 131,468 to 99,020 and a further drop after that [7,22]. This apparent improvement in numbers is credited to regulation enforcement especially in poultry. However, other reasons, such as the deepening of the worldwide economic crisis, could logically influence notification rates from individual states. In accumulative reports, small variations are affected by small annual changes in the numbers reported from individual states. Thus, the reported increases in notification rates from an estimated 21.5 cases to 22.2 per 100,000 people could not only be the result of regulation [7,22,26-28]. It could also result from substantial increases in true infections in individual nations.

Highest notification rates were reported from the Czech Republic, Slovakia and Lithuania (≥70 per 100,000) and the lowest from Portugal, Greece and Romania (≤5 per 100,000) [7]. The last three countries are among those hardest hit by the current economic crisis and the ones reporting the highest hospitalization rates (>85%) due to *Salmonella* infection. Higher hospitalization rates in economically weaker states could result from a combination of reasons, including consumption of unsafe (unregulated) food products. Unfavorable economic conditions couldn’t but affect enforcement of regulations (inability of testing) and of incentives to producers for producing safe products. Lower consumption suppresses pork prices, forcing producers to relax quality control in various ways, including cheaper feed stuffs and labor, lowering cleaning and disinfection costs, keeping animals for longer periods of time in facilities, etc., thus increasing the risks to consumers.

A recession also affects the quality of health care [29,30], thus, early detection and effective treatment of infected consumers, hospitalized only with the worsening of their untreated or improperly treated infection. Thus, higher hospitalization rates in states experiencing economic hardship [7] show, perhaps, the negative impact and the spiral effect of economic distresses on the health of consumers, rather than a better state health care system detecting and reporting hospitalized cases.

On average, 45% of confirmed *Salmonella* cases across EU were hospitalized in recent years having a 0.12-0.14% case-fatality [7,27]. The fatal cases are difficult to treat infections, associated to immunocompromised individuals from other existing diseases, or, perhaps, bad nutrition and stress from economic hardships. Regardless, they are a socioeconomic burden for economically hard-hit public health systems, also having to treat multi-resistant to antimicrobials strains of *Salmonella* spp. deriving from carrier animals [31-33].

Regardless, however, of actual reductions in reported confirmed cases, the pig remained an important contributor to human salmonellosis, responsible, in some states, for up to 56% [34] of the confirmed human cases, with an overall estimated contribution between 10% and 20% [28]. Farmers relaxing control measures during hard to face economic losses seek affordable means for protecting their animals, among which is treating animal cases rather by removing them from their herd. Thus, producing eventually either resistant *Salmonella* strains or increasing the number of subclinical carrier animals.

Subclinically infected animals maintain the microorganism on the farm, contaminating their environment, infecting susceptible animals and contaminating carcasses during slaughtering, eventually passing virulent serovars to consumers. In addition, subclinical infections cause reductions in expected productivity from reduced feed conversion rates, thus lower body weight. Body weight losses are estimated by Danish authorities to about 3 kg per pig [35]. These losses are calculated with a seroprevalence below 10% observed only in nations enforcing effective control programs [36,37]. In nations without control programs or reluctantly enforcing them, pig seroprevalence is higher, affecting productivity and increasing animal treatment costs. The accumulated costs of human cases and pig productivity losses are estimated above €600 million for the EU trading area, €90 million of which result from removing contaminated pig food products from the shelf [6]. The public health estimated costs for EU are €86 million per year or €600-800 per human reported case and rising [9,10,38]. Costs per human case occur from costs investigating and treating cases, but also losses from decreased worker productivity. These estimations are calculated from reported notification rates, but the ability of EU nations to fully investigate, thus accurately record and report human cases depends on each state’s ability to pay the costs. Are, therefore, notification rates accurate? It remains to be systematically investigated.

Thus, economic difficulties, which negatively affect public health and farmers’ income, affect also the investigation of *Salmonella* serovar prevalence, affecting knowledge accumulation on the distribution of virulent serovars in individual nations and eventually the EU.

### Serovars of *Salmonella* spp. infecting pigs and man

#### Salmonella spp. pathogenicity

*Salmonella* spp. are Gram-negative, facultative anaerobic, straight rods, with peritrichous flagella. The genus, having more than 2,600 serovars, is divided into two species, *Salmonella enterica* (the type species) and *S. bongori*. The previously known subspecies of *S. enterica* referred to as subspecies Ι, ΙΙ, ΙΙΙa, IIIb, IV and VI are now respectively named *S. enterica* subsp. *enterica*, *S. enterica* subsp. *salamae*, *S. enterica* subsp. *arizonae*, *S. enterica* subsp. *diarizonae*, *S. enterica* subsp. *hountanae* and *S. enterica* subsp. *indica* (subspecies VI). The majority of the isolated serovars belong to subspecies *enterica*, previously known as subspecies I [39].

The genus *Salmonella*, recovered from most vertebrates and many insects, is considered a “universal pathogen” [40]. However, this term does not imply that all of its serovars are particularly pathogenic for their hosts. It rather means that members of the genus have been isolated from virtually all vertebrates and most reptiles regardless of their virulence. In these hosts, the pathogenicity of various *Salmonella* isolates varies and its expression depends on the ability of the infecting serovar to adapt or not to its host’s biological environment. The consequence of this adaptation is the expression of various degrees of pathogenicity. Hence, a serovar could be very virulent for some hosts and mildly virulent or completely avirullent for others. Among the many thousands of serovars, the most adaptive are serovars Typhimurium and Enteritidis, thus, they are the most frequently isolated from clinical and subclinical infections. If serovars are organized into groups according to their virulence, three groups will be distinguished [41,42].

One including serovars associated with systemic disease in a limited number of hosts, such as *Salmonella* Dublin and *Salmonella* Choleraesuis, another with highly host adapted serovars, such as *Salmonella* Typhi, *Salmonella* Gallinarum and *Salmonella* Abortusovis and a third of all the non-host adapted serovars, known also as unrestricted serovars [41-44].

The above named serovars of the first group are important for man, cattle and pigs. Animals infected with serovars of this group, frequently become “symptomless excreters,” thus are environmental health hazards for susceptible animal species.

The group of highly host adapted serovars, also called “host-restricted serovars,” includes serovars exclusively causing systemic disease to their host, as do serovars Typhi, Gallinarum and Abortusovis in humans, fowl and ovine respectively (HR), thus, considered of less importance to other animal species [44].

However, the group of non-host adapted serovars (unrestricted), among which are the most prevalent in human and animal infections *Salmonella* Typhimurium and *Salmonella* Enteritidis, is the most important group for public health. Serovars of this group infect a wide range of hosts, if the opportunity is given to them, and although they are usually associated with a relatively mild enteric disease, they are the main contributors to state financial losses, as previously mentioned [43,44]. They have the potential to affect animal productivity, and they put a financial burden on a national health system investigating potential human infections.

The ability of a pathogenic serovar to cause disease in an animal species depends largely on the degree of adaptation it exhibits in its host’s biological environment. This ability also defines a serovar’s persistence in the host. An example is serovar Choleraesuis, a porcine adapted serovar, which, although it does not cause the severest disease in swine compared to man, it persists in pig populations making them the reservoir for this serovar [41].

Thus, effective control of animal salmonelloses aims firstly to the protection of consumers and secondly to the maintenance of animal productivity. Both, however, need documentation of serovar prevalence and knowledge of their epidemiological importance before the costs and source of infection are accurately calculated. Farm animals held for mass production of food products, particularly poultry and pigs, are targeted for *Salmonella* control with public health in mind. Documentation of the prevalence of serovars infecting animals helps explain their presence in man and helps formulate the most effective means of restricting their passing from animals to man. It also helps the accumulation of information effectively helping the mapping of the history of *Salmonella* subspecies and serovars in nature.

#### Salmonella spp. infections of pigs and man

In the past, as already mentioned, the most prevalent serovars causing clinical disease to pigs were *Salmonella* choleraesuis, *Salmonella* Typhimurium and *Salmonella* Typhisuis. Each caused a distinct disease syndrome. *S*. Choleraesuis, and particularly variant Kunzendorf, a H_2_S-producing serovar, caused the majority of pig septicaemias [45]. The host unrestricted serovar *Salmonella* Typhimurium, causes acute or chronic enterocolitis, while the rather fastidious serovar *Salmonella* Typhisuis, a host restricted serovar, causes a variety of clinical conditions. Serovar Typhisuis was frequently isolated from cases of mild chronic diarrhea, necrotic colitis, caseous lymphadenopathy, histiocytic interstitial pneumonia and suppurative bronchopneumonia. Although they are very mild clinical manifestations, they are major contributors to animal wasting and loss of productivity [45]. During the years, however, a change in the prevalence of serovars is developing, some of which, although of minor economic importance today, they could become important in the future. Such serovars, isolated with increased frequency from pig feces and pig carcasses, are Dublin, Enteritidis, Copenhagen, Derby, Rissen, Newport, Anatum, Mikawasima, Mbandaka, Agona, Infantis, Οhio, Brandenburg, Virchow, Livingstone and London [46,47]. The importance of these epidemiological observations in animals could fully be revealed, if similar epidemiological investigations were systematically performed in man, when digestive system problems are investigated.

Retrospective reviews of data reveal, however, that during the 50s and 60s *Salmonella* Choleraesuis, rarely isolated from pigs across Western Europe and elsewhere today, was the predominant virulent serovar [48,49]. Today, the prevalence of serovar Typhimurium and specifically the monophasic *S*. Typhimurium, although with a varied between countries prevalence, is consistently emerging as a health risk to consumers [50]. The monophasic serovar Typhimurium in 2011 was the third most frequently reported serovar from pigs, pork products, but also human hospitalized cases. EU reports between 2007 and 2009 showed serovars Typhimurium (63.1%) and Enteritidis (28.3%) by far the commonest serovars from human cases compared to serovars such as Derby (1.9%), Infantis (1.5%), Newport (0.8%) and others [51].

But, as the epidemiological importance of *Salmonella* Choleraesuis in pigs changed the past 50 years in favor of serovars Typhimurium and Enteritidis, less important serovars, Copenhagen, Derby [3,52], Infantis, Virchow and others [53], could become increasingly important replacing eventually the current ones. Thus, because human serovar prevalence follows the one in animals [54], surveillance programs set by the EFSA Panel on Biological Hazards (BIOHAZ) [27] view all *Salmonella* serovars as potential zoonotic pathogens [55,56]. By documenting the genus *Salmonella* as a potential zoonotic pathogen, states and producers are called to spend for consumer protection, through control of animal salmonelloses. Control of animal *Salmonella* infections reduces costs for treating human cases and losses from lost animal productivity and removal of contaminated products from the shelves. Successful control, however, depends on the recognition of existing risks helping the spreading of virulent serovars through animal and human populations, thus, the measures of inhibiting their action.

### Risks during pork production

Pork production is affected by a variety of risk factors among which are quality of pig feed, number of subclinical carriers within a farm, conditions during transportation and lairage premises before slaughtering, slaughter line contamination by carrier animals, conditions during processing and retailing of pork products, conditions of handling pork products during catering and home-food preparation [32,57-59]. Apparently, the control of animal salmonelloses, thus reductions in human infections, is multifaceted and difficult in its implementation without collaboration between the various players and most importantly financial support from state authorities and farmers.

For understanding the way risks in pork production are minimized, one must understand how a pig becomes a *Salmonella* carrier maintaining the infecting serovar in the farm and the retailing. Pigs are infected via the fecal - oral route (feed-water) [32] and rarely via the respiratory tract [60]. The two most important factors for the establishment of a pig infection are the infectious dose and, as previously mentioned, the virulence of the infecting serovar [61]. In the presence of the two, the pathogen becomes established in the gastrointestinal tract and is excreted by the feces, mostly in the absence of clinical disease [62]. Asymptomatic carriers become an important risk to food safety either by intermittently shedding *Salmonella* or having it in several of their tissues and passing it to other stages of pork production [32]. In the farm’s environment, under favorable temperature and humidity, some serovars survive and multiply for almost a year and others, such as choleraesuis, for more than 2 years [63,64]. Serovar survival in the farm puts at the risk susceptible farm, wild and cold-blooded animals. Animal subclinical infections, including pig infections, are contributing to the infection of man either by contact or infected food consumption. To avoid, therefore, this vicious cycle, having in mind human protection, one must control the pathogen from reaching its animal hosts. In the pig farm, the aim is eliminating factors promoting the maintenance of the pathogen within the farm, thus, the infection of susceptible animals either on the farm, during transportation, lairage, and slaughtering.

Some of the measures for lowering the risk to consumers are of low cost and easily implemented. Others are more costly or needing wider surveillance, thus depend on state intervention. Therefore, there is not “one control system” effective for controlling *Salmonella* in all the points of pork production neither is possible to aim and maintain a zero prevalence of the pork production system. When a state’s economic situation permits, it should impose certified pork production for the entire food chain [65]. Otherwise, partially imposed measures become ineffective, thus money losers. State certified pork, promoting the concept “from farm to fork”, a new concept in pig production, depends on the strict enforcement of certain guidelines. Such guidelines, firstly formulated by WHO in the 80s [66], are aiming at controlling the pathogen at the three most important stages of pork production; controlling *Salmonella* infection on the farm, called pre-harvest control, during lairage and slaughtering, called harvest control and during pork product processing and consumer handling, called post-harvest control.

Effective control of pig *Salmonella* infections for the benefit of the consumer depends on good knowledge of the risk factors in each of these three stages. Farmers and farm, transportation, and slaughterhouse workers are those best placed to prevent pork contamination and consumer infection. The first step for promoting the concept “from farm to fork” in pork production is educating those involved in the meat industry on the measures needed for a safer pork product. This concept, internationally promoted through trade agreements and regulations, is the building block of Condex Alimentarious [21] inspiring to regulate the global food trade. Globalized food trade regulations, sounding sensible for the safety of consumers, could be the end of national food security if small national food producers are forced to close by inability to meet set safety rules. Open border trading unions, should be critically scrutinized by citizens, food producers, and ethical politicians. They may eventually realize that the best defence against the loss national food security is a safety, but state sponsored educational programs, educating those involved in meat production on the risks to safety at each production stage. Thus, below are briefly mentioned the most important risks helping the establishment of the pathogen in a host and the best available means of avoiding them at each stage of production.

### Pre-harvest Control of *Salmonella* spp.

The risk of infection for the consumer of pork meat and products should be eliminated if pig infections were eliminated. Although “universal pathogens” cannot be eliminated, they can surely be controlled, if potential risk factors are effectively controlled. These risks are largely associated with pig feed, use of antibiotics, concurrent infections, the size of the herd, farm cleaning practices, floor type, the number of carrier pigs or other animals, such as rodents, birds and insects and their contact with healthy pigs [57-59,67]. Thus, farmers will promote safety if they are educated on how and where to intervene for risk reduction.

*Salmonella* serovars are usually introduced in a pig herd by subclinically infected replacement stock, pigs for fattening entering “open” herds, wild (rodents - birds) or domestic (cats) carrier animals entering the farm’s environment [58,68-70] and even insects (flies) and dust mechanically transferring *Salmonella* serovars from neighboring contaminated environments [71]. However, almost half of pig infections (an estimated 46.7%) result in contaminated feed and water [72,73]. Thus, first aim should be the protection of feed and water from fecal contamination.

Feed staffs are safe, when they are heated for a minimum of four minutes at temperatures above 85°C and when they maintain a moisture content of about 15%. Few feed mills can ensure such conditions, but even if they were ensured, feed stuffs should further be effectively protected from the feces of birds and rodents during cooling, storing and distribution [2,74]. Water should also be free of the pathogen. Water contamination is best avoided if it is supplied with water nipples and not bowls or drinking troughs exposed to fecal contamination [47,75]. Thus, at greater risk of infection are smaller farms or those of nations badly hit by unfavorable economic conditions forcing farmers to cut costs, many times at the expense of feed quality.

Regardless of how a *Salmonella* serovar is introduced in a pig herd, infection will be established in the individual pig, if the pathogen adapts to the host’s gut. Gut content is a good nutritional substrate for microbial multiplication, if also gut osmolarity, pH and mucin production are favorable. Colonization of the host’s gut by the pathogen, thus pathogen survival, is favored by a low digestive tract acidity, inhibiting the multiplication of non-pathogenic microbes acting antagonistically to the establishment of *Salmonella* in the gut [76]. Liquid feeds, particularly fermented ones, produce lactic and acetic acids favoring the multiplication of non-pathogenic microbes. They, restricting the colonization of the gut by virulent serovars, also restrict pathogen survival [77,78]. Whey, cheaply available from the dairy industry [79] and non-pelleted coarse (particle size 903 μm) feed stuffs exhibit a significant protection against *Salmonella* [57,59, 80-83]. Feeds with a particle size 903 μm, compared to finely grounded ones (particle size 639 μm), do not only lower gastric pH, they also slow gastric passage [84,85] giving double protection against the pathogen; inhibiting the pathogens to colonize it and become established, but also minimizing its dissemination.

Dissemination of the pathogen to susceptible animals is further inhibited by the type of the farm and farm flooring type and the density of the pig population. Pigs from finishing farms are 2.5 times more likely to be infected compared to pigs from farrow-to-finish farms. In a farrow-to-finish farming system, there is restricted movement of animals, thus, a minimal risk of mixing healthy with subclinically infected pigs [47]. In a continuous flow system, the risks of infection increase each time weaners are introduced [59]. The same accumulative risk to infection is present when breeder herds keep sows for several years [24], eventually supplying other farms with undetected carrier animals. Seroprevalence among sows is twice as high that of finisher pigs (76.6 vs. 35.4% respectively) [47]. Hence, the rate of infection should increase if fattening pigs remain on contaminated premises for longer than the required time in the hope for a price improvement. This increases the chances of infection due to the spreading of the pathogen through carrier animals or mechanical transfer from older to younger stock. Apparently, an all in-all out system, allowing cleaning, disinfection and the use of sanitary gaps between batches of production, are significantly more effective in minimizing batch cross-infections compared to a continuous system [85].

Equally important is the stocking density. High stocking density statistically increases the risk of infection, because more animals come to contact with potential subclinical carriers. Enlargement of a herd for increasing production outputs, as is the case in “factory type of farming” [86,87], involves a larger number of pig suppliers and a larger number of workers, both increasing the risk of pathogen introduction and dissemination [59,71,88].

In these farms, the type of flooring at holding boxes is another important risk factor. Slated floors slow pathogen dissemination compared to solid or partially slatted floors. The first minimize the bacteriological load and are associated to lower seroprevalence [78]. Frequent emptying of the pit below the slatted floor during lactation reduces further the number of *Salmonella* positive pigs [81].

Therefore, there are various critical points at farm level for risk reduction, some of which are of no or low cost, like the above, but having a long term positive effect. The identification of critical points for interventions and their effectiveness depend, as already mentioned, on a farmer’s knowledge to good management practices. Farmers having a family type pig farm are the usual losers from imposed costly regulations and their state’s chosen indifference during recession times to their problems. Thus, left alone to face problems, they could benefit the most from education on *Salmonella* risk reductions. Their continuous education could be the least costly choice, but having the biggest positive political effect for a state. Continuous education of those involved in meat production of small national economies participating in free trade unions could at the very least sustain national meat production during a recession. If farmers understand how best they can restrict a pathogen’s entrance in their farm and most importantly recognize the potential risk subclinical carriers pose to the health of animals and the consumer, they will protect their livelihood and national food security. Hence, the enforcement of strategies for minimizing or inhibiting the establishment of *Salmonella* pathogenic serovars on an individual host or farm depends greatly on knowledgeable farmers, who must be educated on tax payers’ money, to safeguard the long-term national interests of food security.

### Strategies for Restricting the Establishment of Pathogenic Serovars in Individual Pigs

Strategies for minimizing the establishment of the pathogen in a pig, continuously exploited by researchers, are useful if their costs are significantly lower than their benefits. These strategies are targeting the gut’s defenses. They could work best if an effective vaccine were commercially available and protective against most of the pathogenic serovars [89]. A vaccine, used at all stages of pig production for best results, should not interfere with the serologic monitoring of a farm, when control programs are in effect [90]. In the worst economic situation, an effective vaccine for routinely vaccinating breeding stock and specifically sows, could prevent vertical transmission of the pathogen [91]. Attempts to commercially produce either attenuated or inactivated effective vaccines, and new technology vaccines, have never seized.

Experimentally, the best protection is observed with live attenuated vaccines. They produce better cellular immune response and mucosal IgA production [89,90] conferring good protection against host-specific serovars and to a lesser extent for non host-specific. Ineffective protection against non host-specific serovars, could, however, lead to a type of selective pressure promoting the spreading of serovars, having today minimal epidemiological importance, but becoming a future threat. They also run, under field conditions, the risk of virulence reversion and they show a reduced immune response against non-host specific serovars. They also need proper transportation and storage, safe handling, withholding of antimicrobial treatments before and after their administration and, of course, they may affect daily weight gain due to a brief pyrexia [92].

A safer and economic alternative to attenuated are inactivated vaccines [79], among which are homologous, thus herd-specific *Salmonella* vaccines. They significantly reduce fecal shedding, apparently reducing horizontal transmission [93], for a cost estimated at only $0.85/pig [94]. They, however, offer, like attenuated, effective immunity for few serovars, allowing many others to become future threats. The problems from the use of attenuated and inactivated vaccines, favor the experimental exploitation of new technology vaccines [79]. Molecular vaccines are composed of selected virulence determinants common to many of the pathogenic serovars. Examples of such determinants are purified recombinant proteins, synthetic peptides or plasmid DNA. They show promising immune protection for most of the recognized pathogenic serovars [79]. They are also promising candidates for the production of Differentiating Infected from Vaccinated Animals (DIVA) vaccines, thus vaccines DIVA [95]. The last is a major advantage when serological screening of farms is used for their microbiological confirmation in control programs. DIVA vaccines could become major economic tools of animal protection helping economically smaller farmers to meet the demands of mandatory regulations or economically weak nations, affected by border free trade of animal products, to successfully control this pathogen. Until they are commercially produced, thus cost effective, the well-informed farmer has other alternatives for risk reduction. Undoubtedly, a vaccine effective against most of the *Salmonella* serovars, protecting most of the susceptible animals, could gradually minimize the economic and political importance of *Salmonella* spp. Scientists work toward a successful, universally effective vaccine, which could help in the control of farm animal infections, but it cannot be a panacea against the pathogen. With or without a vaccine, the nature of the pathogen and its natural distribution show that continuous and systematic surveillance of its serovars in food production is a necessity.

Feed acidification by the addition of volatile fatty acids and/or the addition of whey [58,96,97] does not only improve feed conversion and pig growth, but also inhibits pathogen multiplication. A diet having, for the duration of the fattening period, 0.4% lactic and 0.4% formic acids was found to significantly reduce the seroprevalence of *Salmonella* [98,99]. Its drawback is the rising of costs by 2.49 euros per pig [100] and the possible risks from the development of acid tolerant clones of *Salmonella* [101] and clones becoming undetected [102] during field investigations. Exploited cheaper natural alternatives for increasing the defense mechanisms against *Salmonella* infections are the addition in feed of 10% dried sugar beet pulp or the feeding of silage having low concentrations of ammonia, basic amino acids, and biogenous amines [103]. They are also more cost effective for smaller pig herds if these byproducts are locally available from the agricultural industries.

Alternatively, competitive exclusion of the pathogen in the gut is high in the scientific agent at recent times for the reduction of *Salmonella* prevalence. Specifically, the attachment and/or multiplication of *Salmonella* spp. on the gut surface is restricted, either by the multiplication of a non-pathogenic microflora or by the exogenous introduction of such microflora through the feeding of pre- and probiotics. Pre- and probiotics take over gut receptor sites, restricting their access to pathogenic bacteria, thus inhibiting attachment, survival and multiplication of potential pathogens. They also produce antibiotic-like substances or deplete gut substrates essential to pathogen’s survival and multiplication. Thus, they exhibit a nutritional and sanitary effect, becoming a promising and attractive alternative to the in-feed use of antibiotics [104]. Their protective role increases further when production practices in a particular farm are of high standards. High farming standards maintained by knowledgeable farmers, protect the viable counts of the fed beneficial microorganisms, which are more effective if they are pig associated [105]. Beneficial bacteria with significant anti-bacterial activity against *Salmonella* are species of *Streptococcus* and *Lactobacillus*, such as *Streptococcus gallolyticus* subsp. *gallolyticus*, *Streptococcus* *alactolyticus, Lactobacillus reuteri, Lactobacillus delbrueckii, Lactobacillus animals, Lactobacillus salivarius*, *Lactobacillus ruminis* and *Lactobacillus murinus* [106]. Nevertheless, although experimental trials give promising results, more research is needed before they are used as cost-effective treatments for pig salmonellosis [79,105].

Regardless of the means implemented for reducing the risks of infection, the general health of a pig is a critical determinant of its resistance to *Salmonella* infection. Concurrent infections, such as porcine reproductive and respiratory syndrome, correlate positively with higher *Salmonella* prevalence [58,81]. The same is observed with the profuse use of antimicrobial agents to prevent or control various other pig infections, but helping the development of antibiotic-resistant *Salmonella* serovars [107,108]. Pigs, fed a combination of chlortetracycline, procaine penicillin, and sulphamethazine during finishing, were found four times more seropositive to *Salmonella* spp. than those using a probiotic, as a preventive measure [80,103,107]. Apparently, the strategies for minimizing the risk of a pathogenic serovar are analogous to each farmer’s knowledge and attitude.

This farmer must know that salmonellosis, a multi-factorial infection, requires a multi-level approach for its control between and within herds, as well as between and within pigs [2,74]. Therefore, on-farm *Salmonella* control is a major part of the integrated quality control Systems a farmer should know and use for successfully protecting the public from *Salmonella* spp. [58]. The ultimate goal of the pork industry, regardless of size of production, should be the prevention of *Salmonella* serovars entering pig herds, thus the food chain. Measures improving biosecurity, such as the routine testing of new entrees, the lowering of stocking densities and the undertaking of beneficial management practices, protect also from many other important pathogens [109,110], but are most effective in the hands of well-informed pig farmers and farm, transportation and slaughterhouse workers. These people are the key to reductions in product contamination, thus increased consumer’s safety. None, however, of these measures are without actual financial restrains and limitations. Thus, elimination or minimization of pig infections is impossible without a generous state investment toward the education of farmers and all others involved in meat and meat products production. If this important state contribution and responsibility are neglected, as is apparent in economic recessions, free international commerce will overtake national production, thus the control of national food security.

Farm production is the supplier to business of food product development and distribution. Its extinction will affect jobs and cultural eating habits politically affecting the wellbeing of future generations. Such qualitative costs cannot be precisely calculated for small national economies and are not in the agenda of the robust economies of large trading areas, aiming in expending. Farmers of large trading unions remain also outside a small state’s reach for action in cases of broken rules in other stages of production, supplying ready for consumption pork products. Pork products from common commerce areas are offered certified as ready for consumption, thus not routinely investigated for safety in the receiving market. They will be investigated only if public health problems are traced back to a specific pork product. Thus, commercial agreements in border free trading areas, favor the influential makers of safety rules disadvantaging in some ways small national producers of smaller economies needing to comply with the agreed rules.

## Harvest Control of *Samonella* spp.

The effective elimination or minimization of harvest carcass contamination depends mainly on rules protecting the welfare of animals and the proper training of slaughterhouse workers. Stress during transportation of *Salmonella* carrier pigs increases the number of infected pigs. Catecholamines, released during stress, decrease gastric acid production, thus the acidity of the digestive system, helping the survival and multiplication of *Salmonella* and the increasing of peristaltic intestinal movement, thus excretion of *Salmonella* spp. in the environment [111]. Prolonged transportation, under stressful conditions, increases the risk of new pig infections, thus of carcass contamination during slaughtering and processing [112,113]. At this stage, *Salmonella*-free pigs could become carriers, if mixed with existing carriers or sheltered in contaminated premises or transferred on trucks not cleaned and disinfected properly between uses [2]. Withholding of feed for 12-18 h maximum before transport reduces defecation, thus fecal contamination of premises, new carriers and eventually carcass contamination [114]. Exceeding, however, this time of fasting may have the reverse effect [115].

Evidence has shown that, a *Salmonella*-contaminated lairage area is a greater risk to carcass contamination than an infected gut or lymph nodes [116]. Thus, cleaning and disinfection of lairage premises, although it may not completely eliminate contamination [117], it minimizes the risk to it. This risk is further minimized if animals are housed on slatted flooring clean from feces. The importance of lairage premises in carcass contamination demands continuous Hazard Analysis Critical Control Point (HACCP) monitoring [116]. Regular qualitative microbial monitoring of these premises shows the effectiveness of cleansing and disinfection methods [2]. The safety of these premises is further ensured when pigs from high-risk herds are transported, housed and slaughtered after pigs from low-risk herds [118].

The importance of transportation and lairage premises on carcass contamination is revealed in investigations comparing isolates from carcasses before evisceration with those in tissues. *Salmonella* isolates present before evisceration differed from those isolated from the mandibular lymph nodes, evidently presumed that carcass contamination originates mainly from lairage [110,119] and the residual microflora on slaughtering equipment (e.g. carcass splitter) [120-122]. Nevertheless, evisceration and incision of the mandibular lymph nodes are also sources of carcass contamination [123]. Thus, carcass decontamination is the only safe means to either eliminate or effectively reduce carcass cross-contamination, through careless handling, dripping of contaminated water, residual microflora of equipment or evidence of bacteria survival during scalding [124-127]. The temperature of the scalding water or the increased presence of organic matter increases the risk of carcass contamination. Singeing at 1,300-1,500°C is also reducing surface carcass contamination, but it is not very effective against *Salmonella* present in hair follicles [114]. Regardless of actual source for carcass contamination by *Salmonella*, decontamination is effective, if hygiene measures in a slaughterhouse, aiming to minimize the risk from shedders at this stage are meticulously enforced at all critical control points [128]. This protection largely depends on knowledgeable and dedicated slaughterhouse workers handling animals, carcasses and effectively performing regular decontamination of premises.

Three methods of decontamination are applied around the world: (1) Application of hot-water at a temperature of 80°C for 15 s, (2) Hand-held steam vacuum deactivating bacteria in areas of the carcass showing visible fecal contamination and (3) Use of steam ultrasound system killing bacteria at a steam temperature of 130°C and a 30-40 kHz ultrasound [129,130]. Each one of these methods has advantages and disadvantages, and all require an enhancement of their effect by cooling. After decontamination, carcasses must remain in the cooling room for more than 50 h before processing and retailing, because low temperatures and the decline in water activity due to air flow in the cooling room, have a killing effect against *Salmonella* [131].

However, which decontamination method or other interventions during slaughtering are chosen depends on the availability of capital, the cost of energy input, the price of water and the cost of labor [130]. Decontamination costs are calculated to about 1-2% of the total plant costs or €0.19-€0.26 per carcass [129], with the highest cost attributed to hot water decontamination and the lower when a steam vacuum is used [130]. Although costs appear low, smaller facilities or facilities in low-income communities and states cannot, perhaps, afford to have even this low cost added to costs of *Salmonella* control. On the other hand, those having the financial resources can further drop costs of harvest control of *Salmonella*, by selecting and slaughtering serologically negative animals separately from serologically positive. This strategy is low cost when a pre-harvest control program is in effect. Regular surveillance and farm record keeping helps in choosing the right slaughterhouse or manage the time of slaughtering suspect animals from serologically positive farms [6].

The accuracy of serology depends on the sensitivity of the chosen method and specifically the prevalent serovars in an investigated area. It is dependent on the antigenic relatedness of serovars [132] and the site (tissue) colonized by the pathogen [133], but it is a useful tool if carefully chosen for surveillance. Therefore, records kept during surveillance could be used to choose the most effective slaughtering strategy. Thus, costs incurring during pre-harvest and harvest control of *Salmonella* are consolidated, if the two sectors exchange vital information having in mind consumer safety. The benefits to both are even larger if protection measures are expended to include other microorganisms important for public health or important to other stages of pork processing.

## Post-harvest Control of *Salmonella* spp.

A carcass free of important pathogens leaving the slaughterhouse ensures safety of pork products. Manufacturing and retailing of *Salmonella*-free pork products largely depend on the quality of raw materials. This quality is enhanced by the storing of products at temperatures below 7°C, which are inhibiting the growth of *Salmonella*. Further decontamination is also possible by acidification of products, fermentation, curing and smoking, but they are most effective, if the raw material is either *Salmonella* free or having very small loads of pathogens, in general [2]. At post-harvest, there isn’t much flexibility for further increasing safety if the raw material is coming from high-risk farms or slaughter premises.

Thus, consumer safety at all stages of production depends on the status of a pig when leaving the farm or of a carcass when leaving the abattoir. A state committed to the safety of its citizens must effectively intervene at the farm first protecting the food chain and the consumer. This intervention will be against its national interest, if it is based only on punishments, because foreign product suppliers, protected by agreements for free commerce, sale their products on their national certification rules and they are responsible for punishing their farmers. Therefore, the national interest of small economies is best served when punishments come together with incentives for their national farmers producing affordable consumer products of competitive quality. However, incentives have an economic cost and punishments multiple political effects, regardless if they aim farmers or retailers.

Retailers are directly related to consumers and, if there is not a reliable tracing system to connect their product with its specific primary source, punishments to regulate retailers are disadvantaging national retail trade. Thus, again international suppliers of ready to consume pork products in border free commerce areas are advantaged compared to national retailers. Furthermore, tracing the problems at retailing to their actual source, when comes to products from other nations, is of no use in ensuring food safety and quality, as various food scandals have shown [134-136]. These types of investigations do not prevent or improve human *Salmonella* prevalence if punishments and incentives in border free trade are not centrally regulated and financed regardless of the size of business. Because they are left to national agencies of individual states, they should look firstly after the welfare and economic wellbeing of their national producers and resist the pressures of trading partners. Partners are imported competition, which seems initially as benefiting consumers of low income, but after extermination of national competitors they will regulate the food market, and the rules applied according to their interests.

Enforcement of HACCP rules through unrealistic punishments without state paid incentives, when consumer demand decreases, as in an economic recession, will either close smaller producers in favor of larger ones [18] or the final product will not be of the quality regulations demand [137]. Both come with wide national political consequences and economic problems, which should be well considered by individual states in unions promoting commerce between open borders, and in the name of consumer safety, impose on individual states mandatory regulations not considering their economic ability to enforce them. Thus, what is the impact of an economic recession on the control of pig salmonellosis in economically weak states trading under border free conditions?

## Outcome of Synergy between Pig *Salmonella* spp. Infections, Border Free Markets and Economic Recessions

Evidently, the main obstacles to effectively control bacterial zoonoses, such as salmonelloses, are financial rather than scientific or technical. Knowledge and technology are now widely available to producers, regardless of location or size of the facility they manage. They are summarized in WHO regulations [66], trade union regulations [7], Codex Alimentarious [21] and, perhaps, other international agreements to come soon. What is not available to all in enforcing codes and regulations is capital to purchase, replace and maintain technology and thus knowledge. Low consumption lowers prices, thus a producer’s income and his ability to use knowledge and technology for the benefit of consumers. Low product and higher energy costs and prices of feed ingredients increasing the cost for fattening pigs and breeders are decreasing the profits of farmers. Between 2007 and 2008 feeding costs rose due to global price increases in raw materials and remain high, adversely affecting intensive pig farming [6]. Lower feed prices are achieved only by big producers buying in bulk for privately owned feed mills and decontamination units. Smaller producers are subjected to fluctuating prices. If they are forced to comply with regulations controlling the emergence and spreading of pathogens, such as *Salmonella* spp. without the benefit of state incentives, especially during economic recessions, they will close down. The same will result from the high prices of privatized energy sources and water. They are currently on the pressure of privatization across the world including the EU in the name of free trade and globalization eventually adding to the price of food [19,138]. A beneficial state incentive could be to fight for lower prices of energy (electricity and petrol) and water. Those deciding upon these issues when participating in unelected forums and consortiums, as are most of the EU agencies, should keep in mind that the future of their national food security may be at stake [137]. State action should consider the long-term national and livestock economic interests, agricultural security policy, food national security and show sensitivities about consumer safety not only through food safety, but also affordable food availability [20,139]. In the long term, international conditions, developing in a chaotic international political system shadowing sinister actions, disadvantage small nations, which could eventually lose not only their national production systems for the benefit of their nationals, but also their commercial existence, if their politicians give into promises of foreign to them authorities.

Economics, financial inputs/outputs, couldn’t but influence the size and the quality of interventions promoting food safety. Choices made must be cost effective under a highly competitive consumer environment demanded by free trade and international political interests. In an open trade market, losers are those unable, under an economic crisis like the present, to effectively implement control measures for securing their national interests. They are small, local meat producers, having high production costs and competing with lower prices offered by international competitors, facing extinction. These competitors, perhaps, highly helped through state incentives, compete unequally for markets in smaller states unable to subsidize the safety of their pork production. If there is a time, a state should adopt a strategy of incentives for maintaining a low *Salmonella* prevalence or better product quality; that is when consumers restrict their spending affecting the national industry’s income. At such times, national authorities should view the control of pathogens, such as *Salmonella* spp., as a rather political issue involving the consumers’ health and price satisfaction, but also the economic safety of local producers and distributors. Such a dual purpose objective will be successful only through incentives promoting the production of *Salmonella-*free pork and through them pork and pork products free of other important microbes at affordable prices. One important additional reason for such a strategy is that pork meat (and poultry) is, compared to beef meat, more affordable for people, thus its safety and retailing price affects a larger number of people.

Any economic recession poses a challenge to the pork industry worldwide, but mostly for those strangling to produce high quality products at the lowest possible cost. The need to cut costs is evident even in states such as Denmark, having as high priority consumer safety, but also needing to cut the costs for ensuring this safety. Denmark cut these costs from $14 million per annum [140-142] to $8.5 million [100], but at a time previous control efforts had lowered the prevalence of the pathogen [6]. When the prevalence of such a pathogen is effectively lowered, the benefits are lower than the costs, thus costs are cut and states minimize their intervention on behalf of the consumer. Thus, the current border free market conditions in the EU do not favor centrally paid help for individual state food safety, because economic and politically robust member states have already increased the safety of their national products. This is why regulatory actions, although mandatory and centrally decided, are left on the hands of only those who afford their enforcement, thus benefit free commerce. Will they effectively protect the health of consumers, when they eliminate smaller national competitors? This question will correctly be answered, perhaps, in many decades from now.

At times of a recession those unable to support their farmers are small economies of wide border free trading areas, such as the EU. Some Member States, such as Denmark and the Scandinavian countries, in particular Sweden and Finland, have extensively used incentives for monitoring *Salmonella* in pig farms. By choosing this strategy, they aim not only in maintaining a low *Salmonella* prevalence, but also further reducing its prevalence on low risk farms promoting internationally their products taking advantage of border free trade. Similar programs, but with smaller state support, are enforced in the UK, France, Germany, Spain and Italy, all pig meat and meat products producers.

Eastern European countries, however, do not have a consistent history of monitoring and managing *Salmonella* infections perhaps due to continuous economic hardships. They are, thus, good examples of the negative effects economic recessions have on the promotion of nationally produced food under the pressures of free trade. They, economically unable to give incentives, e.g. paid testing of animals before an effective control program is initiated, are leaving the safety of products in the hands of their farmers. They are, however, in the name of free trade, obligated to punish their farmers and retailers when unsafe products reach consumers.

Thus, *Salmonella* control at the present economic and political international environment, regardless of the stage of production, is rather left to the initiative of producers. They must see the benefits and spend accordingly. This is why they should keep in mind that costs for reducing potential hazards, such as *Salmonella*, help also in the reduction of other pathogens of public health importance or product quality [142]. Thus, even small, local producers must, for protecting their future and the interest of future national consumer generations, focus on specific, low cost production weaknesses, to cost-effectively improve pork safety, hence keep their clients until consumption and prices increase. They should remember that *Salmonella* control measures at the pre-harvest stage of production increase the production of pork by about 2.9 pounds per square foot of finisher space (Gorton 2000), increase safety at the harvest stage and help their nation to lower human and animal treatment costs. Thus, although state incentives help local producers during economic recessions and punishments are closing them down, their interests are best served, if they willingly enforce at least those measures with the smallest cost [143] demanding at the very minimum from their national governments free, continuous education. A knowledgeable farmer is a contributor to a consumers’ safety.

## Conclusions

The thousands of serovars, their varied pathogenicity for the various animal species and the difficulties to stimulate effective vaccinal immunity for most of the potential pathogenic serovars, are the main contributors to the economic and political importance of *Salmonella* spp.

This pathogen and similar others are the reasons consumers of border free trading areas demand certification of food safety. They demand it through political pressure on their national governments, which on conditions of free trade use their power to formulate the nature of cross-border regulations. Under free trade, economically robust nations couldn’t but look after their trade expansion. Mandatory regulations for the safety of food products of animal origin, although protective for many millions of consumers, are also indirect means of promoting the national interests of powerful nations.

*Salmonella* spp. pig infections successfully controlled only if surveillance and risk reduction are aiming at pre-harvest, harvest, and post-harvest stages are infections that could be used for consumer product promotions or trade restrictions serving national interests. Therefore, they need taxpayers’ money for control and national trade protection. The stage of production needing the highest money input of *Salmonella* spp. control is the pre-harvest (at the farm). A pig farmer, who has invested in pig feed, buildings, medications, equipment and personal work, unable to meet his financial responsibilities to others and the state due to product price reductions, but higher costs of production, cannot but close its premises in favor of his competitors and against the national food security.

Punishments, low consumption lowering product prices, high energy inputs and even imposed higher income and property taxes extinct national producers leaving their market share to international giants. They are initially offering lower, affordable prices and regulated consumer safety, but no one can really guaranty the same benefits to consumers when local competition is extinct.

The outcome of synergy between pig salmonellosis, free trade and economic recessions in a globalized market of food products is now developing. From the various food safety scandals, the massive increases in individual pig farm outputs (factory pig farming) and the uncontrollable quality (true or false) of available public information (safe - unsafe food), one could conclude that future food issues will be used for or against national interests.

Those able to bear the costs of promotion and food safety is included in promotions, either by truly regulating or effectively hiding their problems will easily overtake foreign consumer markets. All others will leave their national interests exposed to free trade. Can an economically weak nation resist the power of globalization in commerce, thus globalized food problems?

This is a very political question left for answering to those involved in each nation’s affairs. Among them are those businessmen inspiring to become larger and local governing bodies and lobbies giving in to either pressures or bribes. Big nations and their business favored by globalization will increasingly use safety of trading food products to promote their national interests. With the loss of the primary food producing sector in smaller nations, one should expect loss of the food processing sector and eventually loss of cultural eating habits.

Thus, regulations such as those forming Codex Alimentarious, attempted from ancient times to become a tool for dominance, has emerged today as a very powerful globalized tool of food safety control, thus, food dominance. It, and other international trade regulations to come, appear rather codes of promoting globalized trading interest, than codes protecting individual consumers or saving cultural eating habits. They, by promoting factory farming products as safer and affordable, indirectly contribute to the extinction of farming communities, thus increases in urbanization and eventually to consumer total dependence on mass food production, food promised quality and promised lower food prices. Recessions can’t but affect adversely all the above, thus billions of urbanized free trade dependant peoples. Thus, pathogens, such as *Salmonella* spp., could, in the name of safety, easily regulate the behavior of recession hit states on behalf of international business interests.

## Authors’ Contributions

GE collected information and prepared the initial version of the manuscript as part of the literature review of her PhD thesis work, concerning pig salmonellosis. ARB, SK and GC as principal supervisor and co- guides of the PhD Study incorporated valuable suggestions for the conception, planning and correction of the manuscript. All authors read and approved the final manuscript.

## Competing Interests

The authors declare that they have no competing interests.

## References

[1] Acheson D.W, Keusch G.T, LaMont J.T (1997). Intestinal infections with *Salmonella* and *Yersinia* species. Gastrointestinal Infections: Diagnosis and Management.

[2] Lo Fo Wong D.M.A, Hald T, van der Wolf P.J, Swanenburg M (2002). Epidemiology and control measures for *Salmonella* in pigs and pork. Livest. Prod. Sci.

[3] EFSA (European Food Safety Authority) (2010a). The community summary report on trends and sources of zoonoses, zoonotic agents and food-borne outbreaks in the European union in 2008. EFSA J.

[4] Galanis E, Lo Fo Wong D.M.A, Patrick M.E, Binsztein N, Cieslik A, Chalermchaikit T, Aidara-Kane A, Ellis A, Angulo F.J, Wegener H.C (2006). Web-based surveillance and global *Salmonella* distribution, 2000-2002. Emerg. Infect. Dis.

[5] Su L.H, Wu T.L, Chiu C.H (2014). Decline of *Salmonella enterica* serotype Choleraesuis infections, Taiwan [letter]. Emerg. Infect. Dis.

[6] FCC, Food Control Consultants Ltd Consortium (2010). Analysis of the costs and benefits of setting a target for the reduction of *Salmonella* in slaughter pigs for European Commission Health and Consumers Directorate-General SANCO/2008/E2/036 Final Report.

[7] EFSA (European Food Safety Authority) and ECDC (European Centre for Disease Prevention and Control) (2014). The European Union Summary Report on Trends and Sources of Zoonoses, Zoonotic Agents and Food-borne Outbreaks in 2012. EFSA J.

[8] Majowicz S.E, Musto J, Scallan E, Angulo F.J, Kirk M, O’Brien S.J, Jones T.F, Fazil A, Hoekstra R.M (2010). The global burden of nontyphoidal *Salmonella* gastroenteritis. Clin. Infect. Dis.

[9] FCC, Food Control Consultants Ltd Consortium (2011). Analysis of the costs and benefits of setting a target for the reduction of *Salmonella* in breeding pigs for European Commission Health and Consumers Directorate-General SANCO/2008/E2/056 Final Report.

[10] Scharff R.L (2012). Economic burden from health losses due to foodborne illnesses in the United States. J. Food Protect.

[11] Mousing J, Thode Jensen P, Halgaard C, Bager F, Feld N, Nielsen B, Nielsen J.P, Bech-Nielsen S (1997). Nation-wide *Salmonella enterica* surveillance and control in Danish slaughter swine herds. Prev. Vet. Med.

[12] Davies P.R, Funk J.A, Morrow W.E.M (1999). Fecal shedding of *Salmonella* by a cohort of finishing pigs in North Carolina. Swine Health Prod.

[13] SANCO Workshop on *Salmonella* Control in Pigs 26 February 2009.

[14] EC Commision Regulation No 2160/2003 of the European Parliament and of the Council of 17 November 2003 on the control of *Salmonella* and other specified food-borne zoonotic agents. Official J. Eur. Union, L.

[15] EC Commission Regulation No 2073/2005 of November 2005 on microbiological criteria for foodstuffs. Official J. Eur. Union L.

[16] EC Commision Regulation No 1441/2007 of 5 December 2007 amending Regulation (EC) No 2073/2005 on microbiological criteria for foodstuffs. Official J. Eur. Union L.

[17] Hossain N, Byrne B, Campbell A, Harrison E, McKinley B, Shah P (2011). The impact of the global economic downturn on communities and poverty in the UK.

[18] Centre for Retail Research (2014). http://www.retailresearch.org/whosegonebust.php.

[19] The Social Justice Committee (SJC) (2003). Water, Land and Labour: The Impacts of Forced Privatization in Vulnerable Communities. Halifax Initiative Coalition.

[20] von Braun J, Díaz-Bonilla E (2008). Globalization of Food and Agriculture and the Poor.

[21] Winickoff D.E, Bushey D.M (2010). Science and power in global food regulation: The rise of the codex alimentarius. Sci. Technol. Hum. Values.

[22] EFSA (European Food Safety Authority) (2012a). Scientific report of efsa and ECDC. The European union summary report on trends and sources of zoonoses, zoonotic agents and food-borne Outbreaks in 2010. EFSA J.

[23] King R.P, GéBackus B.C, van der Gaag M.A (2007). Incentive systems for food quality control with repeated deliveries *Salmonella* control in pork production. Eur. Rev. Agric. Econ.

[24] EFSA (European Food Safety Authority) (2010b). Scientific opinion on a quantitative microbiological risk assessment of *Salmonella* in slaughter and breeder pigs EFSA panel on biological hazards (BIOHAZ). EFSA J.

[25] Prendergast D.M, Duggan S.J, Gonzales-Barron U, Fanning S, Butler F, Cormican M, Duffy G (2009). Prevalence, numbers and characterizations of *Salmonella* spp. on Irish retail pork. Int. J. Food Microbiol.

[26] EFSA (European Food Safety Authority) (2008a). Scientific opinion of the panel on biological hazards on a request from the European commission on a quantitative microbiological risk assessment on *Salmonella* in meat: Source attribution for human salmonellosis from meat. EFSA. J.

[27] EFSA (European Food Safety Authority) (2013). cientific report of EFSA and ECDC. The European union summary report on trends and sources of zoonoses, zoonotic agents and food-borne outbreaks in 2011. EFSA J.

[28] Veterinary Laboratories Agency (2010). Quantitative microbiological risk assessment on *Samonella* in slaughter pigs and breeder pigs: Final Report.

[29] AAFP (American Academy of Family Physicians) (2009). National Survey of Family Doctors Shows Recession Takes Startling Toll on Patients.

[30] Sussman J.B, Halasyamani L.K, Davis M.M (2010). Hospitals during recession and recovery: Vulnerable institutions and quality at risk. J. Hosp. Med.

[31] Helms M, Ethelberg S, Mølbak K (2005). International *Salmonella* typhimurium DT104 infections, 1992-2001. Emerg. Infect. Dis.

[32] Boyen F, Haesebrouck F, Maes D, Van Immerseel F, Ducatelle R, Pasmans F (2008). Non-typhoidal *Salmonella* infections in pigs: A closer look at epidemiology, pathogenesis and control. Vet. Microbiol.

[33] EFSA (European Food Safety Authority) (2009). Joint opinion on antimicrobial resistance (AMR) focused on zoonotic infections. EFSA J.

[34] EFSA (European Food Safety Authority) (2012b). EFSA panel on biological hazards (BIOHAZ). Scientific opinion on an estimation of the public health impact of setting a new target for the reduction of *Salmonella* in turkeys. EFSA J.

[35] Lo Fo Wong D.M.A, Hald T (2000). *Salmonella* in pork (SALINPORK): Preharvest and Harvest Control Options based on Epidemiological, Diagnostic and Economic Research. Final Report, Contract No: FAIR1 CT95-0400.

[36] Gorton S.J, Kliebensten J, Beran G, Baum D (2000). Economic analysis of *Salmonella* impacts on swine herds. Staff General Research Papers, Iowa State University, Department ofEconomics ASL-R1702.

[37] McNamara P.E, Liu X, Miller G.Y (2003). The Costs of Human Salmonellosis Attributable to Pork: A Stochastic Farm-to-Fork Analysis. Paper prepared for presentation at the American Agricultural Economics Association Annual Meeting, Montreal, Canada, July 27-30, 2003.

[38] Roberts J.A, Cumberland P, Sockett P.N, Wheeler J, Rodrigues L.C, Sethi D, Roderick P.J (2010). The study of infectious intestinal disease in England: Socio-economic impact. Epidemiol. Infect.

[39] Lin-Hui S, Cheng-Hsun C (2007). *Salmonella* Clinical importance and evolution of nomenclature. Chang Gung Med. J.

[40] Fedorka P.J, Cray T, Gray J.T, Wray C, Wray C, Wray A (2000). *Salmonella* infections in pigs. *Salmonella* in Domestic Animals.

[41] Kingsley R, Baumler A.J (2000). Host adaptation and the emergence of infectious disease: The *Salmonella* paradigm. Mol. Microbiol.

[42] Evangelopoulou G, Kritas S, Govaris A, Burriel A.R (2013). Animal salmonelloses: A brief review of “host adaptation and host specificity” of *Salmonella* spp. Vet. World.

[43] Clarke R.C, Gyles C.L, Gyles C.L, Thoen C.O (1993). Salmonella. Pathogenesis of Bacterial Infections in Animals.

[44] Uzzau S, Brown D.J, Wallis T, Rubino S, Leori G, Bernard S, Casadesus J, Platt D.J, Olsen J.E (2000). Host adapted serotypes of *Salmonella enteric*. Epidemiol. Infect.

[45] Griffith R.W, Schwartz K.J, Meyerholz D.K, Straw B, Zimmerman J.J, D’Allaire S, Taylor D.J (2006). Salmonella. Diseases of Swine.

[46] Botteldoorn N, Heyhdrickx M, Rijpens N, Grijspeerdt K, Herman L (2003). *Salmonella* on pig carcasses: Positive pigs and cross contamination in the slaughterhouse. J. Appl. Microbiol.

[47] Vico J.P, Rol I, Garrido V, San Roma N.B, Grillo M.J, Mainar-Jaime R.C (2011). Salmonellosis in finishing pigs in Spain: Prevalence, antimicrobial agent susceptibilities, and risk factor analysis. J. Food Protect.

[48] EFSA (European Food Safety Authority) (2006a). Opinion of the scientific panel on biological hazards on the request from the commission related to “risk assessment and mitigation options of *Salmonella* in pig production”. EFSA J.

[49] Mueller-Doblies D, McLaren I, Weaver J, Davies R.H (2011). A study of the distribution of *Salmonella* serovars in an integrated pig company. Proceedings Book, 9th International Conference on the Epidemiology and Control of Biological, Chemical and Physical Hazards in Pigs and Pork.

[50] Hauser E, Tietze E, Helmuth R, Junker E, Blank K, Prager R, Rabsch W, Appel B, Fruth A, Malorny B (2010). Pork contaminated with *Salmonella* enterica serovar 4,[5],12:i:-, an emerging health risk for humans. Appl. Environ. Microbiol.

[51] Pires S.M, de Knegt L, Hald T (2011). Scientific/Technical Report Submitted to EFSA: Estimation of the Relative Contribution of Different Food and Animal Sources to Human *Salmonella* Infections in the European Union.

[52] EFSA (European Food Safety Authority) (2008b). Report of the task force on zoonoses data collection on the analysis of thebaseline survey on the prevalence of *Salmonella* in slaughter pigs in the EU, 2006–2007. Part A *Salmonella* prevalence estimates. EFSA J.

[53] Methner U, Rammler N, Fehlhaber K, Rosler U (2011). *Salmonella* status of pigs at slaughter- Bacteriological and serological analysis. Int. J. Food Microbiol.

[54] EFSA (European Food Safety Authority) (2011). Analysis of the baseline survey on the prevalence of *Salmonella* in holdings with breeding pigs, in the EU, 2008. Part B: Factors associated with *Salmonella* pen positivity. EFSA J.

[55] Wollin R (2007). A study on invasiveness of different *Salmonella* serovars based on analysis of the enter-net database. Euro. Surveill.

[56] Volf J, Havlickova H, Hradecka H, Ondrackova P, Matiasovic J, Faldyna M, Rychlik I (2010). Epidemiology and interaction of *Salmonella enterica* serovar derby, infantis and typhimurium with porcine alveolar macrophages. Vet. Microbiol.

[57] Kranker S, Dahl J, Wingstrand A (2001). Bacteriological and serological examination and risk factor analysis of *Salmonella* occurrence in sow herds, including risk factors for high *Salmonella* seroprevalence in receiver finishing herds. Berl. Munch. Tierarztl.

[58] van der Wolf P.J, Wolbers W.B, Elbers A.R, van der Heijden H.M, Koppen J.M, Hunneman W.A, van Schie F.W, Tielen MJ (2001a). Herd level husbandry factors associated with the serological *Salmonella* prevalence in finishing pig herds in The Netherlands. Vet. Microbiol.

[59] Lo Fo Wong D.M.A, Dahl J, Stege H, van der Wolf P.J, Leontides L, von Altrock A, Thorberg B.M (2004). Herd-level risk factors for subclinical *Salmonella* infection in European finishing-pig herds. Prev. Vet. Med.

[60] Oliveira C.J, Carvalho L.F, Garcia T.B (2006). Experimental airborne transmission of *Salmonella* agona and *Salmonella* typhimurium in weaned pigs. Epidemiol. Infect.

[61] Loynachan A.T, Nugent J.M, Erdman M.M, Harris D.L (2004). Acute infection of swine by various *Salmonella* serovars. J. Food Protect.

[62] Boughton C, Egan J, Kelly G, Markey B, Leonard N (2007). Rapid infection of pigs following exposure to environments contaminated with different levels of *Salmonella* τyphimurium. Foodborne Pathog. Dis.

[63] Hutchison M.L, Walters L.D, Moore A, Avery S.M (2005). Decline of zoonotic agents in liquid livestock wastes stored in batches on-farm. J. Appl. Microbiol.

[64] Arrus K.M, Holley R.A, Ominski K.H, Tenuta M, Blank G (2006). Influence of temperature on *Salmonella* survival in hog manure slurry and seasonal temperature profiles in farm manure storage reservoirs. Livest Sci.

[65] Lammerding A.M, Fazil A (2000). Hazard identification and exposure assessment for microbial food safety risk assessment. Int. J. Food Microbiol.

[66] WHO (World Health Organization) (1980). Report of the WHO/World Association of Veterinary Food Hygienists (WAVFH) round table conference on the present status of the *Salmonella* problem (prevention and control), Bilthoven, the Netherlands, 6-10 October. WHO/VPH/81.27.

[67] Pfeiffer D.U (2002). Veterinary Epidemiology- An Introduction. Epidemiology Division Department of Veterinary Clinical Sciences.

[68] Barber D.A, Bahnson P.B, Isaacson R, Jones C.J, Weigel R.M (2002). Distribution of *Salmonella* in swine production ecosystems. J. Food Prot.

[69] Leirs H, Lodal J, Knorr M (2004). Factors correlated with the presence of rodents in outdoor pig farms in Denmark and suggestions for management strategies. NJAS-Wagen. J. Life Sci.

[70] Nollet N, Maes D, De Zutter L, Duchateau L, Houf K, Huysmans K, Imberechts H, Geers R, de Kruif A, van Hoof J (2004). Risk factors for the herd-level bacteriologic prevalence of *Salmonella* in Belgian slaughter pigs. Prev. Vet. Med.

[71] Funk J, Gebreyes W.A (2004). Risk factors associated with *Salmonella* prevalence on swine farms. J. Swine Health Prod.

[72] Harris I.T, Fedorka-Cray P.J, Gray J.T, Thomas L.A, Ferris K (1997). Prevalence of *Salmonella* organisms in swine feed. J. Am. Vet. Med. Assoc.

[73] Davies P.R, Scott Hurd H, Funk J.A, Fedorka-Cray P.J, Jones F.T (2004). The role of contaminated feed in the epidemiology and control of *Salmonella enterica* in pork production. Foodborne Pathog. Dis.

[74] Doyle A (2011). Eliminating *Salmonella* in pigs starts with clean feed.

[75] Feder I, Nietfeld J.C, Galland J, Yeary T, Sargeant J.M, Oberst R, Tamplin M.L, Luchansky J.B (2001). Comparison of cultivation and PCR-hybridization for detection of *Salmonella* in porcine fecal and water samples. J. Clin. Microbiol.

[76] Hedemann M.S, Mikkelsen L.L, Naughton P.J, Jensen B.B (2005). Effect of feed particle size and feed processing on morphological characteristics in the small and large intestine of pigs and on adhesion of *Salmonella* enterica serovar typhimurium DT12 in the ileum *in vitro*. J. Anim. Sci.

[77] Farzan A, Friendship R.M, Dewey C.E, Poppe C, Funk J (2010). Evaluation of the risk factors for shedding *Salmonella* with or without antimicrobial resistance in swine using multinomial regression method. Zoonoses Public Health.

[78] Hotes S, Kemper N, Traulsen I, Rave G, Krieter J (2010). Risk factors for *Salmonella* infection in fattening pigs–An evaluation of blood and meat juice samples. Zoonoses Public Health.

[79] Arguello H, Rubio P, Carvajal A, Annous B.A, Gurtler J.B (2012a). *Salmonella* control measures at farm in swine production. *Salmonella* - Distribution, Adaptation, Control Measures and Molecular Technologies.

[80] Leontides L.S, Grafanakis E, Genigeorgis C (2003). Factors associated with the serological prevalence of *Salmonella* enterica in Greek finishing swine herds. Epidemiol. Infect.

[81] Beloeil P.A, Fravalo P, Fablet C, Jolly J.P, Eveno E, Hascoet Y, Chauvin C, Salvat G, Madec F (2004a). Risk factors for *Salmonella enterica* subsp *enterica* shedding by market-age pigs in French farrow-to-finish herds. Prev. Vet. Med.

[82] Rajic A, O’Connor B.P, Deckert A.E, Keenliside J, McFall M.E, Re-Smith R.J, Dewey C.E, McEwen S.A (2007). Farm-level risk factors for the presence of *Salmonella* in 89 Alberta swine-finishing barns. Can. J. Vet. Res.

[83] García-Feliz C, Carvajal A, Collazos J.A, Rubio P (2009). Herd-level risk factors for faecal shedding of *Salmonella enterica* in Spanish fattening pigs. Prev. Vet. Med.

[84] Smith J.L (2003). The role of gastric acid in preventing foodborne disease and how bacteria overcome acid conditions. J. Food Protect.

[85] Somyanontanagul Ν, Nathues H, Tegeler R, Blaha Τ (2008). Comparison between detecting *Salmonella* spp. by bacteriological method and Real-Time PCR assay in samples from pig herds.

[86] Bayer M (1999). Factory Farming. Economic Advantage or Ecological Disaster? A Look at the Economic and Ecological Aspects of Industrial Swine Production in the United States. 081-62-0782.

[87] Boyle P (2014). Industrial Meat.

[88] Casal J, De Manuel A, Mateu E, Martın M (2007). Biosecurity measures on swine farms in Spain: Perceptions by farmers and their relationship to current on-farm measures. Prev. Vet. Med.

[89] Meeusen E.N, Walker J, Peters A, Pastoret P.P, Jungersen G (2007). Current status of veterinary vaccines. Clin. Microbiol. Rev.

[90] Haesebrouck F, Pasmans F, Chiers K, Maes D, Ducatelle R, Decostere A (2004). Efficacy of vaccines against bacterial diseases in swine: What can we expect?. Vet. Microbiol.

[91] Denagamage T.N, O’Connor A.M, Sargeant J.M, Rajic A, McKean J.D (2007). Efficacy of vaccination to reduce *Salmonella* prevalence in live and slaughtered swine: A systematic review of literature from 1979 to 2007. Foodborne Pathog. Dis.

[92] Husa J.A, Edler R.A, Walter D.H, Hoick J.T, Saltzman R.J (2009). A comparison of the safety, cross-protection, and serologic response associated with two commercial oral *Salmonella* vaccines in swine. J. Swine Health Prod.

[93] Roesler U, Heller P, Waldmann K.H, Truyen U, Hensel A (2006). Immunization of sows in an integrated pig-breeding herd using a homologous inactivated *Salmonella* vaccine decreases the prevalence of *Salmonella* typhimurium infection in the offspring. J. Vet. Med. B.

[94] Miller G.Y, Liu X, McNamara P.E, Barber D.A (2005). Influence of *Salmonella* in pigs preharvest and during pork processing on human health costs and risks from pork. J. Food. Protect.

[95] Leyman B, Boyen F, Parys A, Verbrugghe E, Haesebrouck F, Pasmans F (2011). Application of the DIVA principle to *Salmonella* Typhimurium vaccines in pigs avoids interference with serosurveillance programmes.

[96] Schneitz C, Mead G, Wray C, Wray A (2000). Competitive exclusion. *Salmonella* in Domestic Animals.

[97] van der Wolf P.J, van Schie F.W, Elbers A.R, Engel B, van der Heijden H.M, Hunneman W.A, Tielen M.J (2001b). Administration of acidified drinking water to finishing pigs in order to prevent *Salmonella* infections. Vet. Q.

[98] Creus E, Perez J.F, Mateu E (2005). Effect of an acidified diet on *Salmonella* prevalence during the last term of fattening period.

[99] Creus E, Pιrez J.F, Peralta B, Baucells F, Mateu E (2007). Effect of acidified feed on the prevalence of *Salmonella* in market-age pigs. Zoonoses Public Health.

[100] Friendship R.M, Mounchili A, McEwen S, Rajic A (2009). Critical review of on-farm intervention strategies against *Salmonella*. BPEX/ZNCP.

[101] Theron M.M, Lues J.F.R (2007). Organic acids and meat preservation: A review. Food Rev. Int.

[102] Carrique-Mas J.J, Bedford S, Davies R.H (2007). Organic acid and formaldehyde treatment of animal feeds to control *Salmonella*: Efficacy and masking during culture. J. Appl. Microbiol.

[103] Zheng D.M, Bonde M, Sorensen J.T (2007). Associations between the proportion of *Salmonella* seropositive slaughter pigs and the presence of herd level risk factors for introduction and transmission of *Salmonella* in 34 Danish organic, outdoor (non-organic) and indoor finishing-pig farms. Livest. Sci.

[104] Anadón A, Martínez-Larrañaga M.R, Aranzazu Martínez M (2006). Probiotics for animal nutrition in the European Union. Regulation and safety assessment. Regul. Toxicol. Pharm.

[105] Jacela J.Y, DeRouchey J.M, Tokach M.D, Goodband R.D, Nelssen J.L, Renter D.G, Dritz S.S (2010). Feed additives for swine: Fact sheets – prebiotics and probiotics, and phytogenics. J. Swine Health Prod.

[106] Collazos J.A, Samaniego L.M, García C, de Castro L, Carvajal A, Rubio P (2008). Selection and characterization of anti-salmonella lactic acid bacteria of porcine origin.

[107] Gebreyes W.A, Thakur S, Morrow W.E.M (2006). Comparison of prevalence, antimicrobial resistance, and occurrence of multidrug-resistant *Salmonella* in antimicrobial-free and conventional pig production. J. Food Protect.

[108] Emborg H.D, Baggesen D.L, Aarestrup F.M (2008). Ten years of antimicrobial susceptibility testing of *Salmonella* from Danish pig farms. J. Antimicrob. Chemother.

[109] EFSA (European Food Safety Authority) (2006b). Opinion of the scientific panel on biological hazards on “risk assessment and mitigation options of *Salmonella* in pig production”. EFSA J.

[110] De Busser E.V, De Zutter L, Dewulf J, Houf K, Maes D (2013). *Salmonella* control in live pigs and at slaughter. Vet. J.

[111] Williams L.P, Newelln K.W (1967). Patterns of *Salmonella* excretion in market swine. Am. J. Public Health North.

[112] Hurd H.S, McKean J.D, Griffith R.W, Wesley I.V, Rostagno MH (2002). *Salmonella enterica* infections in market swine with and without transport and holding. Appl. Environ. Microbiol.

[113] Beloeil P.A, Chauvin C, Proux K, Madec F, Fravalo P, Alioum A (2004b). Impact of the *Salmonella* status of market-age pigs and the pre-slaughter process on *Salmonella* caecal contamination at slaughter. Vet. Res.

[114] Berends B.R, Urlings H.A, Snijders J.M, van Knapen F (1996). Identification and quantification of risk factors in animal management and transport regarding *Salmonella* spp. in pigs. Int. J. Food Microbiol.

[115] Martin-Pelaez S, Peralta B, Creus E, Dalmau A, Velarde A, Perez J.F, Mateu E, Martin-Orue S.M (2009). Different feed withdrawal times before slaughter influence caecal fermentation and faecal *Salmonella* shedding in pigs. Vet. J.

[116] De Busser E.V, Maes D, Houf K, Dewulf J, Imberechts H, Bertrand S, De Zutter L (2011). Detection and characterization of *Salmonella* in lairage, on pig carcasses and intestines in five slaughterhouses. Int. J. Food Microbiol.

[117] Swanenburg M, Urlings H.A, Keuzenkamp D.A, Snijders J.M (2001). *Salmonella* in the lairage of pig slaughterhouses. J. Food Protect.

[118] Alban L, Sørensen L.L (2010). Hot-water decontamination - an effective way of reducing risk of *Salmonella* in pork. Fleischwirtschaft.

[119] Kich J.D, Coldebella A, Morés N, Nogueira M.G, Cardoso M, Fratamico P.M, Call J.E, Fedorka-Cray P, Luchansky J.B (2011). Prevalence, distribution, and molecular characterization of *Salmonella* recovered from swine finishing herds and a slaughter facility in Santa Catarina, Brazil. Int. J. Food Microbiol.

[120] Baptista F.M, Dahl J, Nielsen L.R (2010). Factors influencing *Salmonella* carcass prevalence in Danish pig abattoirs. Prev. Vet. Med.

[121] van Hoek A.H, de Jonge R, van Overbeek W.M, Bouw E, Pielaat A, Smid J.H, Malorny B, Junker E, Löfström C, Pedersen K, Aarts H.J, Heres L (2012). A quantitative approach towards a better understanding of the dynamics of *Salmonella* spp. in a pork slaughter-line. Int. J. Food. Microbiol.

[122] Visscher C.F, Klein G, Verspohl J, Beyerbach M, Stratmann-Selke J, Kamphues J (2011). Serodiversity and serological as well as cultural distribution of *Salmonella* on farms and in abattoirs in Lower Saxony, Germany. Int. J. Food Microbiol.

[123] Hald T, Wingstrand A, Swanenburg M, von Altrock A, Thorberg B.M (2003). The occurrence and epidemiology of *Salmonella* in European pig slaughterhouses. Epidemiol. Infect.

[124] Gill C.O, Bryant J (1993). The presence of *Escherichia coli*, *Salmonella* and *Campylobacter* in pig carcass dehairing equipment. Food Microbiol.

[125] Bolton D.J, Pearce R, Sheridan J.J, McDowell D.A, Blair I.S (2003). Decontamination of pork carcasses during scalding and the prevention of *Salmonella* cross-contamination. J. Appl. Microbiol.

[126] Botteldoorn N, Herman L, Rijpens N, Heyndrickx M (2004). Phenotypic and molecular typing of *Salmonella* strains reveals different contamination sources in two commercial pig slaughterhouses. Appl. Environ. Microbiol.

[127] Alban L, Stärk K.D (2005). Where should the effort be put to reduce the *Salmonella* prevalence in the slaughtered swine carcass effectively?. Prev. Vet. Med.

[128] da Silva L.E, Dias V, Ferronatto A, Guerra P, Berno L, Triches N, Kich J.D, Corbellini L.G, Cardoso M (2012). Longitudinal dissemination of *Salmonella enterica* clonal groups through the slaughter process of *Salmonella* -positive pig batches. J. Food Protect.

[129] Lawson L.G, Jensen J.D, Christiansen P, Lund M (2009). Cost-effectiveness of *Salmonella* reduction in Danish abattoirs. Int. J. Food Microbiol.

[130] Baptista F.M, Halasa T, Alban L, Nielsen L.R (2011). Modelling food safety and economic consequences of surveillance and control strategies for *Salmonella* in pigs and pork. Epidemiol. Infect.

[131] Argüello H, Carvajal A, Collazos J.A, García-Feliz C, Rubio P (2012b). Prevalence and serovars of *Salmonella enterica* on pig carcasses, slaughtered pigs and the environment of four Spanish slaughterhouses. J. Food. Res.

[132] Arrach N, Porwollik S, Cheng P, Cho A, Long F, Choi S.H, McClelland M (2008). *Salmonella* serovar identification using PCR-based detection of gene presence and absence. J. Clin. Microbiol.

[133] Nollet N, Maes D, Duchateau L, Hautekiet V, Houf K, van Hoof J, De Zutter L, de Kruif A, Geers R (2005). Discrepancies between the isolation of Salmonella from mesenteric lymph nodes and the results of serological screening in slaughter pigs. Vet. Res.

[134] Irish Exporters Association (2008). Latest information on pork and beef contamination.

[135] WHO (World Health Organization) (2010). Dioxins and their effects on human health. Fact sheet N°225.

[136] Wacker R (2013). The European Food Industry’s Horse Meat Scandal.

[137] Nellemann C, MacDevette M, Manders T, Eickhout B, Svihus B, Prins A.G, Kaltenborn B.P (2009). The environmental food crisis – The environment’s role in averting future food crises. A UNEP rapid response assessment. United Nations Environment Programme, GRID-Arendal.

[138] Garrido A, Calatrava J (2010). Agricultural Water Pricing: EU and Mexico. OECD (Organisation For Economic Co-Operationand Development).

[139] Woods A (2011). A historical synopsis of farm animal disease and public policy in twentieth century Britain. Philos. Trans. R. Soc. B.

[140] Wegener H.C, Hald T, Lo Fo W.D, Madsen M, Korsgaard H, Bager F, Gerner-Smidt P, Molbak K (2003). *Salmonella* control programs in Denmark. Emerg. Infect. Dis.

[141] Wegener H.C (2010). Danish initiatives to improve the safety of meat products. Meat Sci.

[142] van der Gaag M.A (2004). Epidemiological and economic simulation of *Salmonella* control in the pork supply chain.

[143] Goldbach S.G, Alban L (2006). A cost-benefit analysis of *Salmonella* -control strategies in Danish pork production. Prev. Vet. Med.

